# The road to drug resistance in *Mycobacterium tuberculosis*

**DOI:** 10.1186/s13059-014-0520-1

**Published:** 2014-11-13

**Authors:** Anastasia Koch, Robert John Wilkinson

**Affiliations:** MRC/NHLS/UCT Molecular Mycobacteriology Research Unit and NRF/DST Centre of Excellence, Institute of Infectious Disease and Molecular Medicine, Department of Clinical Laboratory Sciences, University of Cape Town, Observatory, Cape Town, 7925 South Africa; Clinical Infectious Diseases Research Initiative, Institute of Infectious Diseases and Molecular Medicine, University of Cape Town, Observatory, Cape Town, 7925 South Africa; MRC National Institute for Medical Research, London, NW7 1AA UK; Department of Medicine, Imperial College London, London, W2 1PG UK

## Abstract

Sequencing of serial isolates of extensively drug-resistant tuberculosis highlights how drug resistance develops within a single patient and reveals unexpected levels of pathogen diversity.

Tuberculosis (TB) remains a crucial public health problem, with increasing drug resistance posing a challenge to current control efforts. Treatment regimens for drug-susceptible TB are onerous, requiring a minimum of six months of treatment with four antitubercular drugs. There are patients who develop multi-drug-resistant (MDR), extensively drug-resistant (XDR) and totally drug-resistant (TDR) forms, which are successively more difficult to treat. In these circumstances, treatment regimens involve the use of a larger number of less-effective drugs, which have a narrower therapeutic margin.

In many bacteria, drug-resistance determinants are carried on mobile genetic elements. However, in *Mycobacterium tuberculosis* (Mtb), drug resistance is exclusively associated with point mutations and chromosomal rearrangements. Poor or intermittent therapy has long been thought to be the major explanation for drug resistance, and it is believed that drug-resistant strains develop through the sequential fixation of a small set of mutations, such that the pathogen samples only a small proportion of possible evolutionary paths [1].

The application of whole-genome sequencing (WGS) has revealed previously underappreciated levels of genetic diversity within circulating Mtb populations, and the implications of this diversity for transmission and disease outcomes are increasingly being acknowledged. By contrast, mycobacterial heterogeneity within a single host, and any concomitant biological or clinical significance, has been explored but seldom documented.

In a study published in this issue of *Genome Biology*, Eldholm and colleagues apply WGS to investigate the evolution from drug-sensitive to XDR-TB within a single patient [2]. This adds to an emerging body of evidence that suggests that intra-host microbial diversity is substantial and might have significant consequences when inferring transmission. There are few instances, if any, in the literature where this has been investigated in such detail.

## Mapping drug-resistant tuberculosis in a single patient

The study team took advantage of nine serial bacterial isolates collected from a single patient, from the time of first diagnosis of drug-susceptible TB through to the development of XDR-TB. The infection ultimately resolved following the addition of linezolid to the treatment regimen - linezolid is known to be a moderately effective treatment for drug-resistant TB when other options have failed [2]. Illumina sequencing was applied to the isolates, which were collected over a period of 42 months, achieving a high depth of coverage. By relaxing the stringency of filters applied to detect single-nucleotide polymorphisms (SNPs), the authors observed unexpected levels of heterogeneity within individual samples. It is worth mentioning that DNA was not extracted from single colonies - instead ‘loopfuls’ of bacterial cells were harvested for DNA. This approach allowed the group to detect SNPs present at a frequency of approximately 25% at sites with a minimum read-depth of 50. Of the 35 SNPs identified in this way, 20 were transient and 15 eventually became fixed. Twelve of the observed mutations were associated with drug resistance, and phenotypic resistance was observed at the same time that genotypic resistance emerged.

Although the patient was infected with only one Mtb strain, multiple resistance alleles were observed for antitubercular drugs throughout the course of infection, with the exception of rifampicin and kanamycin [2]. The levels of micro-heterogeneity reported are consistent with those of previous studies [3, 4].

These observations suggest that, at any given time, there might be significant Mtb diversity within a single patient. High levels of diversity could affect the accurate interpretation of WGS data that are used to infer transmission. Current WGS of Mtb requires subculture of the bacilli from patient samples to ensure that sufficient DNA is available for analysis, and bacterial subpopulations that are not culturable are not captured during downstream sequencing [5]. Moreover, one cannot be certain that a single sputum sample represents all regions of the lung; indeed significant intra-lesional heterogeneity has been observed in humans [6]. Therefore, intra-patient variability could be even higher than reported by the aforementioned studies and might not be detectable by current WGS approaches.

## Advantages of adaptability: survival of the fittest

By virtue of access to multiple samples from the entire course of disease, Eldholm and colleagues were able to calculate mutation rates for the sample set [2]. The results are unique in that they suggest that non-resistance mutations hitchhike with, or link to, resistance SNPs to become fixed within the population. This phenomenon could partly explain the high numbers of SNPs recently found to occur in global collections of drug-resistant Mtb isolates [7].

When the authors removed both resistance and hitchhiking mutations from the analysis, they found that the mutation rate was only slightly higher than that recently reported in cohorts with drug-susceptible disease [8]. Their findings suggest that the selective pressure conferred by antibiotics provides Mtb with an opportunity to diversify and adapt. Drug resistance represents a readily measureable determinant of genetic change because its effects can be attributed to a set of discrete SNPs. There are additional factors whose impact on the genome of Mtb are not well established, such as the vaccination status of individual patients or their status with respect to human immunodeficiency virus (HIV). These factors are much more complicated to consider, but important and interesting nonetheless.

Significantly, Eldholm and colleagues complemented genomic data with phenotypic evaluation of the fitness of isolates with different resistance mutations [2]. Results of growth-rate measurements in the presence of the drug indicated that the fittest strains contained mutations that eventually became fixed. While there may be caveats when assaying Mtb fitness under laboratory conditions, these results provide further support for the notion that resistance mutations with the lowest fitness costs will eventually become fixed within a population.

Additionally, through RNA sequencing of a subset of samples, the authors observed a stable change in transcriptional patterns as drug resistance emerged [2]. Genes that were differentially regulated included those involved in efflux and synthesis of cell-wall components. The targets of antibiotics are, by definition, functionally essential [1], and the changes in transcriptional remodeling could indicate an important mechanism that facilitates adaptation to the fitness costs of having mutations in multiple essential genes [2].

## For the future

The study by Eldholm and colleagues has provided valuable insights into the evolution of Mtb drug resistance within a single host, and in the process it has demonstrated the considerable diversity and adaptability of the pathogen [2]. As any good study should, it also raises important questions for further investigation.

The SNPs identified in the current study were validated by PCR [2] - however, the use of a low threshold to assign a SNP as genuine imparts the possibility of false positives. Additionally, the sequencing of DNA from batches of cells cannot determine the single-cell origin of specific mutations, and therefore epistatic interactions, which have been shown to be important in the evolution of drug resistance in Mtb [9], cannot be investigated by this approach. However, the application of WGS technologies to areas such as culture-independent [10] and single-cell sequencing will provide the necessary tools to investigate these issues further.

Although the phenotypic implications of resistance mutations have been extensively studied [1], the biological and clinical significance of the hitchhiking mutations is largely unknown. Nevertheless, the study by Eldholm *et al*., which was conducted in in Norway [2], is a striking reminder that TB drug resistance can quite easily develop, even within the context of very low TB rates in a well-functioning control program. It will be of vital importance to determine similarly how micro-heterogeneity influences disease outcomes and treatment efficacy in regions where a high burden of TB and elevated infection pressure might result in unique inter- and intra-host-strain competition dynamics [5].

## Abbreviations

HIV: Human immunodeficiency virus
MDR: Multi-drug-resistant
Mtb: *Mycobacterium tuberculosis*
SNP: Single-nucleotide polymorphism
TB: Tuberculosis
TDR: Totally drug-resistant
WGS: Whole-genome sequencing
XDR: Extensively drug-resistant.
